# Perceptions of the Social Relevance of Science: Exploring the Implications for Gendered Patterns in Expectations of Majoring in STEM Fields

**DOI:** 10.3390/socsci6010019

**Published:** 2017-02-21

**Authors:** Sarah Blanchard Kyte, Catherine Riegle-Crumb

**Affiliations:** 1 Education & Employment Research Center, Rutgers University, 94 Rockafeller Road, Second Floor, Piscataway, NJ 00854-8054, USA; 2 Population Research Center, The University of Texas at Austin, 305 E. 23rd Street, Stop G1800, Austin, TX 78712-1699, USA

**Keywords:** social relevance, science attitudes, perceptions, gender, STEM, expectations, majors, field of study, middle school, high school

## Abstract

Despite efforts to increase participation in science, technology, engineering and math fields (STEM), the role of students’ perceptions of the social relevance of science in guiding their expectations to major in STEM remains largely unexplored. Though science education scholars predict that perceptions of social relevance likely matter equally for boys and girls, gender scholars suggest that these perceptions should matter more for girls than boys. Using longitudinal data from a large, urban, low-income, and predominantly minority-serving district, this study examines the potentially gendered role of perceptions of social relevance in ninth graders’ expectations to major in STEM. Further, it examines these dynamics with respect to expectations to major in any STEM field as well as expectations to major in specific STEM fields. Findings largely support the perspective of gender scholars; perceptions of the social relevance of science positively and significantly predict female, but not male, students’ intentions to major in STEM (vs. non-STEM fields). Subsequent analyses that look at intentions to major in specific STEM fields reveal a similar pattern, such that perceptions of relevance positively predict female students’ intentions to major in the biological sciences, the physical sciences, and engineering, while male students’ intentions are not similarly impacted. By contrast, positive perceptions of the relevance of science predict a modest increase in interest in computer science for both boys and girls.

## 1. Introduction

Labor market analysts have been sounding the alarm regarding the need for more workers trained in science, technology, engineering and math fields (STEM) for more than a decade, as the U.S. economy is increasingly reliant on innovation and growth in these fields [1–3]. Researchers in several disciplines have attempted to understand why, despite the high demand and the accompanying high status and income associated with careers in many STEM fields, the percent of students choosing to study these fields in college has remained stagnant, or even decreased in some cases [4]. Additionally, as women remain greatly underrepresented in many STEM fields, there is a large public as well as academic discourse regarding the obstacles to recruiting and retaining female students [5,6]. Of course, the roots of the problem extend far back beyond the labor force and the postsecondary realm, as research indicates that young people make decisions quite early about whether they intend to pursue STEM fields in the future [7,8]. Though the notion of a STEM pipeline has been rightly critiqued for being too simplistic, it is nevertheless clear that adolescents’ early decisions in this regard are highly predictive of their subsequent behaviors. So what factors motivate young people to choose to study STEM fields, and why are female students much less likely to make such choices?

In an effort to understand these dynamics, researchers within the fields of sociology, psychology, and education have concentrated on the role that several different factors, including academic performance, attitudes, and self-perceptions of competence play in both shaping students’ decisions to pursue STEM fields and in creating and sustaining gender inequality [5,8–11]. In general, while recognizing the presence of gender socialization and stereotypes, much of the research in this area can be characterized as arguing that female underrepresentation is driven by the fact that females trail behind their male peers on the academic (e.g., high test scores) or psychological (e.g., self-confidence) factors that best predict entry into STEM fields [6,12]. Such studies have certainly helped to establish a strong foundation of knowledge regarding the factors that do (and alternatively do not) contribute to inequality. Yet, we argue that what is currently lacking are empirical studies that go beyond focusing on individuals’ skills and perceptions of their own abilities and attitudes, and instead focus explicit attention on how young people actually see and make sense of science itself, and how this may have implications for gendered choices. Specifically, while research on gender disparities often assumes (either implicitly or explicitly) that girls’ and boys’ views of science likely play a role in shaping their decisions to later enter such fields [4,13–15], they typically do not attempt to actually measure such views nor investigate their potential impact on the choices that students subsequently make.

To address this shortcoming in the literature, in this study we examine whether and how perceptions of the social relevance of science contribute to male and female students’ expectations of majoring in STEM fields. In doing so we build on insights from gender scholars [9,16,17] as well as those in science education [18–20], as each provides different predictions regarding the role of gender. Specifically, gender theories would predict that, consistent with dominant cultural beliefs about women’s presumed innate preferences, perceptions of science as a domain that has broad applicability for improving human life would be much more important for influencing female students’ decisions to enter STEM fields compared to male students. Yet, research in science education would instead suggest that perceptions of social relevance would be important for the subsequent decisions of both male and female students, as views of science as having meaning and utility for life outside the classroom are thought to be important motivators for all students to want to continue to study and pursue STEM fields. Thus, we will investigate whether both male and female students’ future expectations are similarly positively impacted when they view scientific fields as contributing to the improvement of society, or whether instead, such views are more important in shaping the expectations of female students.

Moreover, our investigation will move beyond considering students’ expectations to pursue STEM fields in the aggregate. Importantly, women’s representation varies quite substantially across fields within STEM, such that a singular focus only on the broad category of STEM can obscure critical differences. Specifically, in 2013, women—who earned 57% of all bachelor’s degrees—earned 59% of degrees in the biological sciences and 39% of those in the physical sciences, but only 19% and 18% of degrees in computer science and engineering, respectively [21]. Our study will examine students’ expectations to major in each of these different STEM fields, and thus reveal whether perceptions of social relevance may be more important in shaping young people’s future plans to pursue certain fields than others [22].

To investigate these issues, we draw on longitudinal data collected in a large, urban, predominantly minority, and low-income school district. As such, the students at the center of our study are set within an educational context that mirrors those inhabited by ever increasing percentages of young people [23]. Additionally, while minority and low-income youth are often underrepresented in STEM fields in college (and beyond), research has documented relatively high STEM interest among such student populations [24,25]. And by moving our attention past the typical focus on predominantly white populations, we gain insight into the expectations of young people who represent the changing demographics of the country.

## 2. Theoretical Framework

### 2.1. Considering the Role of Social Relevance in Shaping Females’ Interest in STEM

Sociologists studying gender inequality generally posit that gender is socially constructed, such that is created and maintained through interactions at the individual level as well as the institutionalization of gendered roles and expectations at the societal level [17]. Cultural stereotypes about gender play a large role in this construction, shaping the expectations and perceptions people have for themselves and for others according to their gender [17,22]. Scholars studying gender inequality within STEM fields in particular tend to concentrate on measuring the existence and impact of stereotypes that are directly STEM-related, such as the view that males are innately better at math than females [22,26]. For example, research by Correll [9] discusses how gender-STEM stereotypes lead girls to doubt their confidence in their own ability (despite high levels of performance), subsequently leading them to be less likely than their male peers to declare STEM majors in college.

In this paper, we argue that while we have learned much from this body of research, we need to focus more explicit attention towards broad gender schemas and stereotypes that may also have implications for gendered patterns in STEM fields. Specifically, Charles and Bradley [27] argue that in contemporary Western societies such as the United States, egalitarian beliefs that all individuals should have equal opportunities in the public sphere, including access to education, exist alongside persistent cultural beliefs that men and women are essentially and fundamentally different [16,28]. Such beliefs are manifest in decisions such as the selection of a college major, where choices reflect societal beliefs about the types of work and activities for which men and women are each presumed to be differentially and innately suited to perform. For example, women are stereotyped as naturally more nurturing and concerned with the well-being of others, while men are presumed to be more individualistic, analytical, and competitive [16].

Importantly, these dominant views about essential differences between the genders also map onto perceptions of different occupational and educational fields. Some gender scholars argue that to the extent that science is not perceived to have direct applicability to helping others and benefiting society as a whole, then a decision to enter such fields would conflict with females’ presumed natural preferences [8,29]. Yet in fact, there is little empirical research that focuses specifically on individuals’ perceptions of science fields, and whether and how they are linked to gendered decisions about whether or not to pursue STEM. While some studies have identified gender differences in preferences for work activities, such as working alone or in teams, and linked this to subsequent gender differences in the likelihood of choosing a STEM field [5,29,30], the extant literature generally stops short of considering individuals’ actual perceptions of science as a domain.

Thus in this paper, building on the insights of gender scholars, we seek to empirically investigate the claim that to the extent that science is viewed as not socially relevant—meaning it is narrow in its application and does not address societal problems—females will be much less likely than their male peers to express an intention to pursue related fields. At the same time, this perspective implies that when students do perceive science as socially relevant, this should hold more sway in increasing females’ interest in pursuing STEM compared to males.

### 2.2. The Role of Social Relevance in Increasing STEM Interest for All Students

Of course those studying gender inequality are not the only scholars whose research focuses on increasing students’ interest in STEM. Researchers in the field of science education have long focused on understanding the factors that lead to student engagement and learning in science and related fields. Classical theorists like Piaget as well as Dewey [18], called early attention to the importance of science classrooms that made explicit connections between the curriculum and the real world, recognizing the need for instruction that emphasized the broad application and power of science to transform human life. In more recent years, educational researchers have again called attention to this issue, arguing that ‘school science’ too often treats science fields as varied collections of abstract historical discoveries and intangible phenomenon, asking students to memorize decontextualized facts and concepts that result in their becoming bored and disinterested [31,32]. Current educational reforms are working to change this [20] and although limited in scope, there is empirical evidence that students who view science as socially relevant are more likely to remain engaged with the content and express interest in continuing to study science [19,31,33–35], and that curriculum that directly emphasizes the broad applications and benefits of science for human life can indeed be effective in promoting all students’ positive views [36].

Thus educational theories as well as empirical research emphasize the power of perceptions of the relevance of science in shaping educational outcomes for all students, regardless of gender. This is not to say that the science education literature is not concerned with gender differences, yet the emphasis is typically on identifying those instances where girls trail behind boys, such as science self-confidence, and then focusing on how these could be improved [37]. And studies that explore gender differences in students’ perceptions of the social relevance of science typically find that girls’ and boys’ views are actually very similar [32,33,38]. As such, research in this area does not typically consider these perceptions to be a likely contributor to gender differences in science interest or related future expectations.

### 2.3. This Study

Stepping back, the insights of two different areas of research offer two essentially competing hypotheses about the impact of perceptions of the social relevance of science on students’ subsequent interest in pursuing future educational opportunities in related fields. Within science education, researchers are very concerned with students’ views of science, yet generally work from the presumption that perceptions of social relevance will equally benefit all students regardless of gender. From this perspective, when both girls and boys view science as applying directly to real life and having the capacity to improve society in a myriad of ways, they will subsequently be more motivated and likely to want to continue to study it. Yet on the other hand, operating from a different theoretical lens, gender scholars call attention to dominant cultural beliefs about essential differences between males and females, which include stereotypes of women as inherently concerned with the well-being of others and the general health of society (and the planet). Thus consistent with normative gender scripts, views of science fields as being socially relevant (or not) should be more powerful in shaping the subsequent decisions of female students compared to male students. In this paper, we will empirically examine these two alternative predictions. Moreover, while women are well-represented in the biological sciences, and to a lesser extent, the physical sciences (with high representation in chemistry, a large field, but not in physics, a comparatively smaller field), they remain vastly under-represented in computer science and engineering fields at the postsecondary level as well as in the labor force [5,21]. Indeed some have argued that the reason behind these gendered patterns of representation is that women perceive computer science and engineering as abstract and disconnected from social concerns, and thus not directly related to helping others [29]. Thus we will examine whether students’ perceptions of social relevance are more or less closely linked to expectations to pursue some STEM majors more than others.

## 3. Data and Methods

Data for this study come from the Broadening Science in School Study (BSSS) set within a large, diverse school district in one of the biggest cities in the Southwest. The vast majority of students in the district qualify for free or reduced price lunch (80%) and the student body is primarily Hispanic (62%) with smaller percentages of Black (25%) and white (8%) students. In many ways, the characteristics of this district are quite common for U.S. school children. For example, a recent study from the Southern Education Foundation notes that 51% of students nationwide are in poverty [39]. Furthermore, within urban settings, 64% of students are eligible for free or reduced price lunch programs suggesting that such disadvantages are the norm rather than the exception [40]. Moreover, school desegregation has stalled (or perhaps even reversed) in recent years, while the share of the school-age population comprised by minority students has dramatically increased [41]. Thus a large share of students in the U.S. currently attend racially and economically segregated schools like those within our focal district.

As part of the BSSS, members of the research team collected survey data from students within this district for several years as part of a larger study aimed at understanding students’ science experiences in school. Administrative data were also collected in the form of students’ academic transcripts (including their grades, test scores, etc.). Finally, students’ demographic data were obtained from the district including their gender, race/ethnicity, and other characteristics such as their eligibility for free or reduced price lunch.

The analytic sample for this study is comprised of a cohort of students who were 8th graders in the Fall of the 2012 academic year, and who then transitioned to high school as 9th graders the following Fall (2013). We limited the sample to those students who reported at least some likelihood that they would attend college (retaining all but 3% of students). Additionally, due to the very small percentages of students who were Asian or identified as ‘other’ race, we chose to restrict our analyses to white, Black, and Hispanic students.^1^ Students who did not complete questions about their expected college major (our dependent variable) were excluded from the sample. Missing data on the independent variables was quite limited (ranging between 0%–6%) and was singly imputed using STATA’s *impute* command. Our final analytic sample includes 935 students attending 13 high schools.

### 3.1. Expectations to Major in STEM

Our dependent variables are constructed from students’ responses to a survey question they answered in the Fall of their 9th grade year that asked, “If you attend college, how likely is it that you would choose to major (or specialize) in each of the following fields?” This question asked students about four STEM-related fields (biological sciences, physical sciences, computer science and technology, and engineering). Student responses were reported on a scale ranging from 1 (not at all likely) to 5 (very likely). As a value of 3 represents a neutral response, we consider students who responded with a 4 or 5 for any of these four fields to be expecting to major in STEM. Our first dependent variable considers STEM expectations in the aggregate and distinguishes between those who expect to major in any of these four STEM fields vs. those who do not. In a second set of analyses we consider each field separately (e.g., distinguishing those who expect to major in the biological sciences from those that do not, those that expect to major in the physical sciences from those that do not, and so on).^2^ A correlation table including all of the dependent variables and the independent variables described below are included in Appendix A (Table A1).

**Figure 1.**
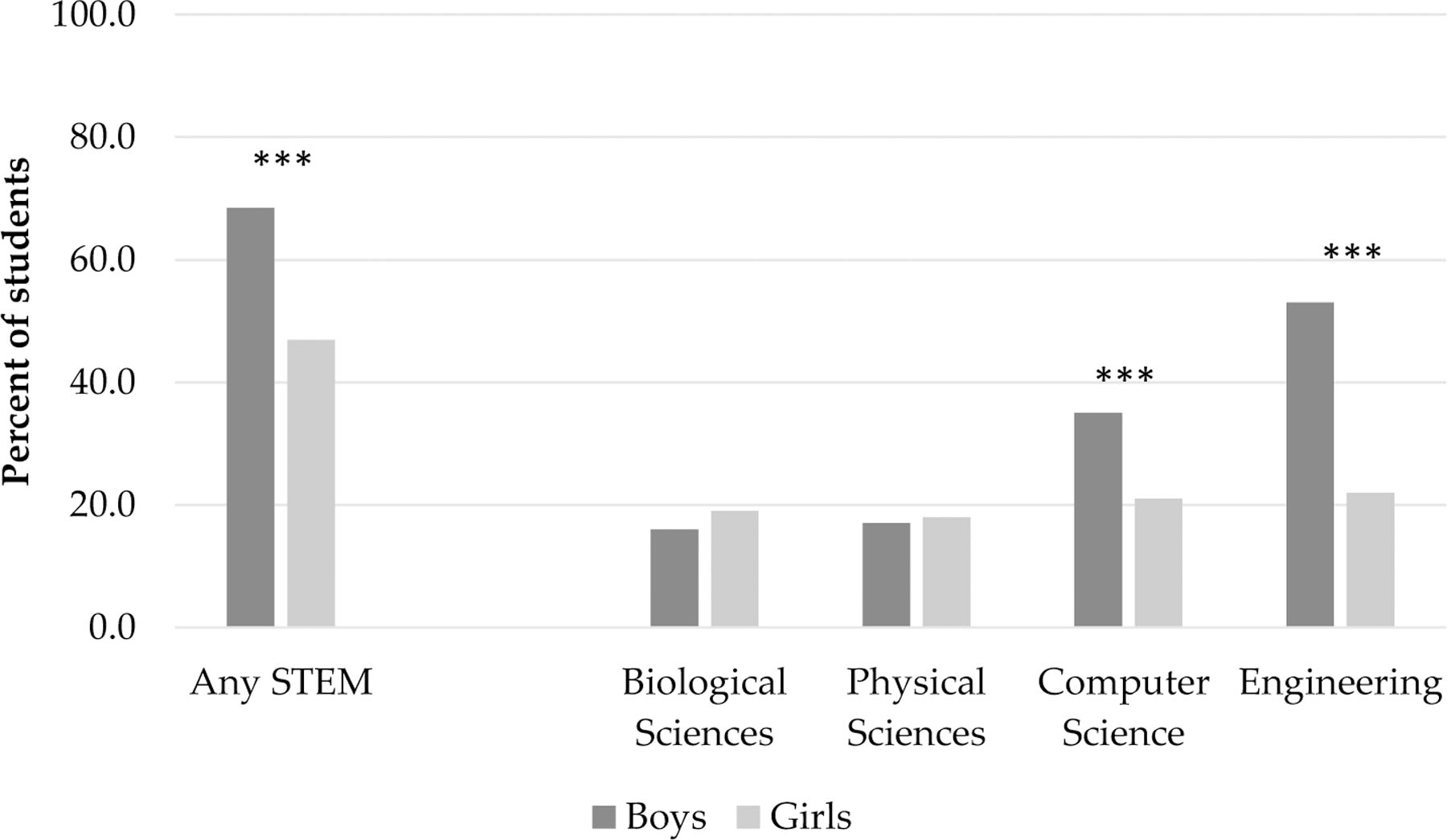
Expectations to major in STEM fields by gender among 9th grade students. *** *p* < 0.001, ** *p* < 0.01, * *p* < 0.05, ~ *p* < 0.10, two tailed test.

As seen in Figure 1, gender differences in expectations to major in STEM are quite striking. The leftmost two bars show that 47% percent of girls report an expectation of majoring in any STEM field compared to 69% of boys. Further, in examining students’ plans to major in specific STEM fields, the right side of Figure 1 shows that field-specific gender gaps in STEM expectations are observable at this age. In particular, boys and girls have similar expectations of majoring in both the biological and physical sciences. Specifically, 16% of boys and 19% of girls in our sample planned to major in the biological sciences whereas 17% of boys and 18% of girls planned to major in the physical sciences (neither difference is statistically significant). Furthermore, consistent with national patterns at the postsecondary level, there are very large and statistically significant gender differences in expectations to major in both computer science and engineering among the adolescents in our sample. Specifically, 35% of boys but only 21% of girls expected to major in computer science, while 53% of boys and only 22% of girls expected to major in engineering.

### 3.2. Perceptions of the Social Relevance of Science

The independent variable at the center of this study is students’ perceptions of the social relevance of science. We utilize a scale comprised of students’ averaged responses in the Spring of their 8th grade year to the following items: “science helps people,” “a lot of people never use science in their lives,” “science is useful for solving everyday problems,” “everyone uses science sometimes,” “I only use science at school,” and “there are all kinds of jobs or careers that use science.” Items were re-coded so that a high score indicated a positive response. Because students’ views of science were largely positive, we dichotomized each item to account for the skewed distribution of students’ attitudes and to distinguish between those who strongly agreed vs. not (agree, disagree, strongly disagree). The Cronbach’s alpha for the scale is 0.71, and an exploratory factor analysis confirms that this scale is unidimensional with similar loadings across the component items. As reported in Table 1, boys and girls had similar perceptions of the social relevance of science (pooled mean = 0.36). This is consistent with other studies that have examined young people’s views of the relevance of science [32–35,38] and underscores the fact that the focus of our study is not about whether girls have a deficit in a STEM-specific resource (as evidenced by a lower mean, for example), but rather whether or not girls’ expectations to pursue STEM are more strongly shaped by their perceptions of the social relevance of science.

### 3.3. Control Variables

#### 3.3.1. Social Background

The multivariate models in this analysis use a set of controls for students’ social background characteristics. These include students’ race/ethnicity (available from administrative data), immigrant status, and a proxy for social class. Immigrant status measures whether students reported in the survey that they were born in a country other than the U.S. (coded 1) or born in the U.S. (coded 0). For social class background, we utilize a proxy that measures the number of books in one’s home (commonly used in international and national studies of this age group including TIMSS). It is a dichotomous variable distinguishing between those who report having enough books in the home to fill one or more bookcases (coded 1) and those who report that their homes have fewer than enough books for one bookcase (coded 0).

Table 1 shows the descriptive statistics for these background variables, both overall and by gender. Consistent with the demographics of the school district, 73% percent of the sample is Hispanic, 10% is white, 17% is Black, and 15% were born outside of the United States. On average, students report having fewer books at home than it would take to fill a bookcase.

#### 3.3.2. Science Achievement

Because prior research finds that students’ academic achievement in STEM fields positively predicts their subsequent intentions to pursue such fields in college [6], our models include a summary measure that captures students’ 8th grade science test score, as well as their science grades and level of course-taking. Specifically, students’ transcripts included their score on the state accountability exam in science, as well as their cumulative grade average in science (originally scaled as 0–100). Transcripts also indicated whether or not the student was in an honors or advanced science course.^3^ To avoid issues of multicollinearity and account for the different scales of each original measure, we created standardized versions of each measure, and then calculated the mean to create a summary measure used in the multivariate models that follow. Consistent with the prior literature in this area [42], girls and boys at this age are not statistically different from one another in their science achievement.

#### 3.3.3. Science Affect

As mentioned previously, there is a large extant research literature examining the influence of gender differences in social-psychological variables on subsequent gaps in STEM fields [9,43]. Therefore, to better assess the potentially unique contribution of our focal variable, perceptions of the social relevance of science fields, our models also take into account students’ own personal feelings towards science. We include a science affect scale, comprised of students’ responses to three items on the 8th grade survey: “I like science,” “science is fun,” and “I enjoy learning science” (Cronbach’s alpha = 0.79). As before, we account for the positive skew by dichotomizing each item to distinguish between those who strongly agree vs. those who do not, before taking the mean across all items. We note here that this measure is only moderately correlated with perceptions of social relevance (R = 0.40). Moreover, boys report significantly more positive affect towards science (0.36) than do girls (0.29).^4^

### 3.4. Analytic Plan

The analysis in this study proceeds in two main parts. First, we examine the extent to which students’ perceptions of the social relevance of science predict their subsequent expectations to major in STEM by conducting logistic regression models predicting students’ likelihood of expecting to major in any STEM field (versus not). The baseline model includes students’ background characteristics, academic achievement in science, and their science affect. The second model adds the measure of students’ perceptions of the social relevance of science to examine whether boys and girls who perceive science as more socially relevant are in turn more likely to expect to major in any STEM field. Finally, to examine whether social relevance may matter more for girls’ expectations, we include an interaction between gender and social relevance in the third model.

In the second part of our analysis, we use the same approach to predict students’ expectations of majoring in each of four STEM fields—namely the biological sciences, physical sciences, computer science and technology, and engineering. Once again, we first examine models with only the main effect of social relevance, and then include an interaction term between social relevance and gender to address whether perceptions of the social relevance of science matter more for girls’ expectations to major in specific fields of STEM. Throughout, we utilize clustered models with robust standard errors that take into account the nesting of students within schools.^5^

## 4. Results

### 4.1. Expectations to Major in Any STEM Field

The first part of our analysis examines whether students’ perceptions of the social relevance of science shape expectations to major in any STEM field and whether these perceptions may be especially powerful for girls. The results are included in Table 2, below. Consistent with prior research [11,44,45] our baseline model (model 1) shows that girls are less likely than boys to intend to major in STEM fields in the aggregate (consistent with Figure 1), while those from higher social class background (as captured through the proxy of books in the home), and students who are born outside the U.S. are also significantly more likely to expect to major in STEM (although the p-value for the former is 0.06 indicating a marginally significant effect). Students with higher levels of science achievement are also significantly more likely to expect to major in a STEM field (although the effect is borderline at *p* = 0.09), as are those with higher levels of science affect. In model 2, we find that students who perceive science as more socially relevant are in turn more likely to expect to major in STEM. This effect is only borderline significant (B = 0.42, *p* = 0.09), and appears weaker than the measure for science affect. Specifically, in model 2, a one standard deviation increase in a students’ perceptions of the social relevance of science is associated with an increase of 0.03 in the probability of expecting to major in STEM, while a one standard deviation increase in science affect results in a predicted increase of 0.07.^6^ As expected, adding perceptions of social relevance to the model does not diminish the gender gap in expectations as boys and girls have similar means.

Model 3 adds an interaction between gender and perceived social relevance to the set of variables previously included in Model 2. The interaction term is positive and statistically significant, indicating a greater effect of relevance for girls. Additionally, the main effect is now negative, smaller, and not significant.

**Figure 2.**
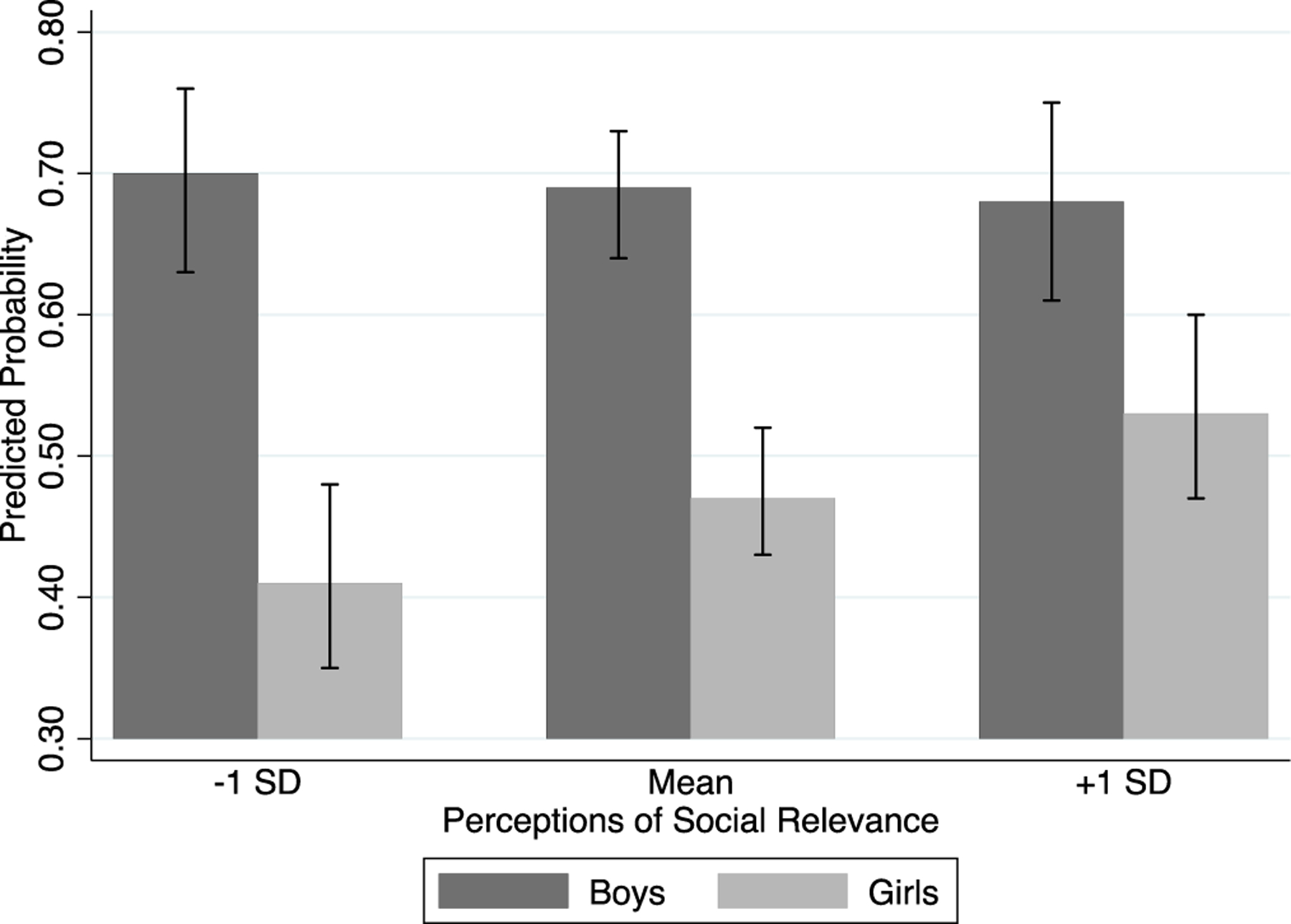
Predicted probability of expecting to major in STEM by perceptions of social relevance and gender.

To better illustrate the gendered patterns from model 3 of Table 2, in Figure 2 we show the predicted probabilities of expecting to major in STEM for boys and girls as a function of their perceptions of the social relevance of science. All other variables in the model are held to the mean. We see that as perceptions of the social relevance of science increase, girls’ probabilities of expecting to major in STEM increase substantially, though boys’ probabilities remain relatively flat. Put differently, while boys’ average probability of having expectations to major in a STEM field is quite high (around 0.7), it is virtually insensitive to their perceptions of science. However, for girls, perceiving science as more socially relevant is associated with a much higher likelihood intending to major in STEM. Thus, students’ perceptions of the social relevance of science clearly operate in notably gendered ways in shaping plans to major in STEM.

### 4.2. Field-Specific STEM Expectations

To address the second part of our research agenda, we now examine whether perceptions of the social relevance of science shape expectations to major in specific fields within STEM and whether and how this may differ by gender. In Table 3, we proceed with the same series of covariates in our models as in Table 2. However, this analysis models students’ likelihood of expecting to major in the biological sciences ([A] models, leftmost section of table), the physical sciences ([B], second section from the left), computer science ([C], second section from the right), and engineering ([D], rightmost section).

Turning first to expectations to major in the biological sciences, the baseline model (A1) shows that students with higher science affect as well as those born outside the U.S. and those with more books at home are more likely to expect to major in the biological sciences. Model A1 also shows that girls are significantly more likely than boys to intend to major in the biological sciences. In the second model (A2) we see that students who perceive science as more socially relevant are significantly more likely to expect to major in the biological sciences. Finally, in model A3, the interaction between gender and social relevance is positive and marginally significant (*p* = 0.08), while the main effect is greatly diminished and no longer statistically significant. Thus the pattern of results for the biological sciences generally follows that observed in Table 2 for any STEM field. Specifically, calculating predicted probabilities (with other variables held to the mean) reveals that as girls’ perceptions of social relevance increase from one standard deviation below the mean to one standard deviation above the mean, their probability of declaring a biological science major increases from 0.13 to 0.24, while boys’ predicted probabilities (non-significantly) increase from 0.13 to 0.15.

Moving to the next set of models within Table 3, we now consider the role of social relevance in shaping expectations to major in the physical sciences. In the baseline model we see that those born outside the U.S., those with more books in the home, and those with a more positive science affect are more likely to expect to major in the physical sciences. Also consistent with Figure 1, we see that gender is not a significant predictor of expectations of majoring in the physical sciences. In model B2, we see a positive and significant effect of perceptions of the social relevance of science. Yet with the inclusion of the interaction between gender and relevance, we once again see that this positive association is driven by girls, as the main effect moves very close to zero and is not significant, indicating no discernible impact for boys, while the interaction is positive and statistically significant. Specifically, as girls’ perceptions of social relevance increase from one standard deviation below the mean to one standard deviation above the mean, their probability of declaring a physical science major increases from 0.11 to 0.22, while boys’ predicted probabilities remain at around 0.15.

The next portion of Table 3 examines the role of social relevance in shaping expectations to major in computer science. As expected, the baseline model (C1) shows that girls are much less likely to expect to major in computer science, while those with higher science affect are more likely. Model 2 reveals a small, positive, and marginally significant effect (*p* = 0.08) of perceptions of social relevance. Unlike previous models, the coefficient measuring the interaction between gender and social relevance in the third model is close to zero and not statistically significant. With its inclusion, the main effect remains virtually the same in size but is no longer statistically significant.

Finally, the rightmost models of Table 3 consider the role of social relevance in shaping expectations to major in engineering. The baseline model (D1) shows that girls are much less likely to expect to major in engineering compared to boys, while those born outside the U.S., high science achievers, and those with higher levels of science affect are significantly more likely than their peers to plan to major in engineering. Including perceptions of science relevance in model D2 explains away the positive effect of science affect found in D1, yet social relevance does not significantly predict students’ expectations to major in engineering. Yet with the inclusion of the interaction term in model D3, which is positive and statistically significant, it appears that the absence of a main effect obscures the positive impact of perceptions of social relevance that exists uniquely for girls. Specifically, as girls’ perceptions of social relevance increase from one standard deviation below the mean to one standard deviation above the mean, their probability of declaring an engineering major rises from 0.17 to 0.26, while boys’ predicted probabilities hover at around 0.5. Taken together, consistent with the biological and physical sciences but in contrast to computer science, girls alone appear more likely to plan to major in engineering when they perceive science to be socially relevant.

## 5. Discussion

Despite decades of scholarly attention on the topic of what draws students to STEM fields, there is a lack of empirical literature that focuses explicit attention on how young people perceive science, and how such perceptions may be directly linked to their future plans to pursue STEM fields. This study attempts to address this by drawing on two different areas of research that essentially present competing ideas about whose intentions may be shaped by views of science as socially relevant. Specifically, gender scholars hold that gender stereotypes guide girls toward fields that are viewed as having the broad capacity to help others, improve life, and make a difference in the world. From this perspective, girls who perceive science as socially relevant may be more likely to pursue these fields as a way of fulfilling the normative feminine role dictated by prevailing cultural beliefs about gender, while boys’ decisions to enter STEM are likely unaffected by perceptions of social relevance, as normative masculine roles do not include placing a priority on such things. By contrast, scholars from within science education would argue that perceiving science as socially relevant encourages STEM expectations similarly among both girls and boys, as students are more likely to want to continue studying a subject that has meaning and importance outside of school walls. This study examines this issue directly by examining whether perceptions of social relevance guide expectations to major in STEM among adolescents. In doing so, we examine not only the role of social relevance in guiding interest in STEM in general, but also the extent to which social relevance guides students toward specific majors within STEM—namely, the biological and physical sciences, computer science, and engineering. Because students’ expectations as ninth graders will guide their decision-making and academic preparation for college, these foreshadow gendered pathways into STEM in higher education and ultimately, the labor force [6,46].

In examining how perceptions of the social relevance of science shape expectations to major in STEM at the start of high school, we found that viewing science as socially relevant clearly increases the likelihood that students will intend to major in STEM in ways that are gendered. Consistent with the view offered by gender theorists, we found highly gendered patterns in how social relevance guides students’ intentions to pursue STEM majors in the aggregate. As seen in Table 2 (and the associated Figure 2), perceiving science as more socially relevant is associated with a statistically significant and substantial increase in girls’ expectations to major in a STEM field, while boys’ expectations are not moved in response to such views. Subsequent field-specific analyses reveal that this same gendered pattern appears in three of the four STEM fields considered, specifically, the biological sciences, physical sciences, and engineering. Thus we find that in our sample, perceptions of social relevance are an important predictor of adolescent girls’ intentions to enter STEM postsecondary fields where women are currently well-represented (the biological sciences, and to a lesser extent, the physical sciences) as well as in engineering, a field that remains highly male-dominated [21]. Indeed, this suggests that recent efforts, such as ad campaigns by organizations such as Exxon/Mobil that highlight the power of engineering to change the world for the better, could perhaps move the needle towards gender equity [47].

By contrast, our results for computer science are not quite as clear. We find a borderline significant main effect of the perceived social relevance of science, and no evidence of a gender interaction. Thus on the one hand, the results might be interpreted as consistent with science education researchers who suggest that all students benefit when they view science as relevant and powerful for solving problems outside of school walls. In practice, this insight offers a potentially useful avenue for increasing participation in computer science by linking it to solving real-world problems in the minds of both male and female adolescents. Yet, we also note that the effect we observe is relatively small, and thus such perceptions may do little on a practical scale to move more girls into a field where women are so grossly under-represented.^7^

## 6. Conclusion

Though the specific focus of our paper was on examining the gendered impact of perceptions of social relevance, our findings also speak to larger conversations about gender gaps in STEM participation. Specifically, we found that a majority of ninth-grade girls in our sample (54%, compared with 32% of boys) have already expressed a disinterest in pursuing any STEM major. Moreover, the gendered patterns we observe in Figure 1 regarding future intentions in specific STEM fields largely mirror current patterns of gender representation in postsecondary education at the national level as well as within the labor force [47]. Thus our results underscore the powerful role of gender in shaping students’ plans long before the transition to college, as well as hint that perhaps we should not anticipate that younger cohorts will play a role in changing patterns of gender segregation. Yet at the same time we note one potentially positive sign; perhaps reflecting changes in interest in technology among younger cohorts, computer science and engineering were the most popular STEM majors for both girls and boys in our sample.

As with any study, there are several limitations and other questions that arise that cannot be addressed by the present study. First, because this study focuses on predominantly Hispanic high school students in one large, urban school district, it is not possible to say how the patterns observed here would compare to other contexts. Future research could address this, as well as whether patterns might be similar or different among both younger and older student populations. Moreover, we looked for but did not find racial/ethnic differences in how social relevance predicted students’ STEM intentions.^8^ Moving forward, it would be informative to consider these dynamics at the intersection of race/ethnicity and gender, which unfortunately we could not explore due to sample size. Furthermore, specific survey items examining how students view the social relevance of specific STEM fields, such as engineering, would be even more informative than the general questions utilized in this study. Current efforts to address such issues are hampered by the scarcity of data that is both large in scope and detailed in its STEM focus. Yet, by demonstrating that perceptions of social relevance may help guide more girls into STEM fields, future scholars can build on the contribution begun here.

## Figures and Tables

**Table 1.** Descriptive statistics for pooled sample and reported separately by gender.

	Overall		Boys		Girls		Sig Dif
	Mean	SD	Mean	SD	Mean	SD	
*Dependent Variables*							
Expects to major in STEM	0.56		0.69		0.47		***
Expected STEM major							
Biological sciences	0.18		0.16		0.19		
Physical sciences	0.18		0.17		0.18		
Computer science	0.27		0.35		0.21		***
Engineering	0.36		0.53		0.22		***
*Focal Independent Variable*							
Perception of the social relevance of science	0.36	0.31	0.35	0.32	0.37	0.31	
*Control Variables*							
Social background							
Race/ethnicity							
White	0.10		0.08		0.10		
Black	0.17		0.18		0.17		
Hispanic	0.73		0.74		0.72		
Born outside of the U.S.	0.15		0.17		0.14		
Books in the home	0.40		0.37		0.42		
Science achievement	0.03	0.77	0.04	0.77	0.02	0.76	
Science affect	0.32	0.39	0.36	0.41	0.29	0.38	*
N	935		407		528		

*** *p* < 0.001,

** *p* < 0.01,

* *p* < 0.05,

~ *p* < 0.10, two tailed test.

**Table 2.** Results from logistic regression models predicting expectations to major in any STEM field (Coefficients with standard errors in parentheses) (N = 935).

	M1		M2		M3	
	Coef	*p*	Coef	*p*	Coef	*p*
*Focal Independent Variable*						
Perceived social relevance			0.42 (0.25)	~	*−*0.14 (0.37)	
*Interaction Effects*						
Female * Relevance					0.93(0.46)	*
*Female*	*−*0.88 (0.14)	***	*−*0.91 (0.14)	***	*−*1.22 (0.22)	***
*Control Variables*						
Social background						
Race/ethnicity						
Black	0.10 (0.30)		0.08 (0.30)		0.09 (0.30)	
Hispanic	0.25 (0.26)		0.27 (0.26)		0.29 (0.26)	
Born outside of the U.S.	0.74 (0.21)	***	0.75 (0.21)	***	0.76 (0.21)	***
Books in the home	0.31 (0.16)	~	0.31 (0.16)	~	0.32 (0.16)	*
Science achievement	0.17 (0.10)	~	0.14 (0.10)		0.14 (0.10)	
Science affect	0.89 (0.18)	***	0.76 (0.20)	***	0.80 (0.20)	***
Constant	0.17 (0.29)		−0.04 (0.30)		0.11 (0.31)	

*** *p* < 0.001,

** *p* < 0.01,

* *p* < 0.05,

~ *p* < 0.10, two-tailed test.

**Table 3.** Results from Logistic Regression Models Predicting Expectations to Major in Specific STEM Fields (Coefficients with standard errors in parentheses) (N = 935).

	Biological Sciences						Physical Sciences						Computer Science						Engineering					
	A1		A2		A3		B1		B2		B3		C1		C2		C3		D1		D2		D3	
	Coef	*p*	Coef	*p*	Coef	*p*	Coef	*p*	Coef	*p*	Coef	*p*	Coef	*p*	Coef	*p*	Coef	*p*	Coef	*p*	Coef	*p*	Coef	*p*
*Focal Independent Variable*																								
Perceived social relevance			0.82 (0.31)	**	0.23 (0.46)				0.75 (0.31)	*	0.03 (0.44)				0.47 (0.27)	~	0.46 (0.35)				0.31 (0.26)		−0.18 (0.34)	
*Interaction Effects*																								
Female * Relevance					0.99 (0.56)	~					1.29 (0.56)	*					0.02 (0.47)						1.01 (0.46)	*
*Female*	0.36 (0.18)	*	0.34 (0.18)	~	−0.09 (0.30)		0.11 (0.18)		0.09 (0.18)		−0.47 (0.30)		−0.66 (0.15)	***	−0.68 (0.15)	***	−0.69 (0.24)	**	−1.39 (0.15)	***	−1.41 (0.15)	***	−1.79 (0.23)	***
*Control Variables*																								
Social background																								
Race/ethnicity																								
Black	0.22 (0.36)		0.19 (0.36)		0.20 (0.36)		−0.14 (0.36)		−0.17 (0.36)		−0.16 (0.36)		0.29 (0.34)		0.27 (0.34)		0.27 (0.34)		−0.40 (0.31)		−0.42 (0.31)		−0.40 (0.30)	
Hispanic	0.24 (0.31)		0.32 (0.32)		0.34 (0.32)		−0.04 (0.31)		0.03 (0.31)		0.05 (0.32)		0.45 (0.30)		0.48 (0.31)		0.48 (0.31)		−0.17 (0.27)		−0.15 (0.27)		−0.12 (0.27)	
Born outside of the U.S.	0.68 (0.22)	**	0.69 (0.22)	**	0.70 (0.22)	***	0.54 (0.22)	*	0.55 (0.23)	*	0.57 (0.23)	*	0.22 (0.20)		0.23 (0.20)		0.23 (0.20)		0.49 (0.20)	*	0.49 (0.20)	*	0.50 (0.20)	*
Books in the home	0.53 (0.19)	**	0.54 (0.19)	**	0.55 (0.20)	**	0.46 (0.20)	*	0.48 (0.20)	*	0.49 (0.20)	*	−0.05 (0.17)		−0.04 (0.17)		−0.04 (0.17)		0.12 (0.17)		0.12 (0.17)		0.13 (0.17)	
Science achievement	0.01 (0.12)		−0.05 (0.13)		−0.05 (0.13)		−0.12 (0.12)		−0.18 (0.13)		−0.18 (0.13)		−0.06 (0.11)		−0.09 (0.11)		−0.09 (0.11)		0.23 (0.10)	*	0.21 (0.11)	~	0.21 (0.11)	*
Science affect	1.08 (0.21)	***	0.85 (0.23)	***	0.88 (0.23)	***	1.33 (0.21)	***	1.12 (0.23)	***	1.16 (0.23)	***	0.54 (0.19)	**	0.39 (0.20)	~	0.39 (0.20)	~	0.38 (0.18)	*	0.28 (0.20)		0.31 (0.20)	
Constant	−2.70 (0.37)	***	−2.99 (0.39)	***	−2.78 (0.40)	***	−2.36 (0.36)	***	−2.62 (0.38)	***	−2.36 (0.39)	***	−1.21 (0.33)	***	−1.34 (0.34)	***	−1.34 (0.35)	***	0.06 (0.29)		−0.02 (0.30)		0.11 (0.31)	

*** *p* < 0.001,

** *p* < 0.01,

* *p* < 0.05,

~ *p* < 0.10, two-tailed test.

## Footnotes

2 Note that the dependent variables measuring students’ field-specific interests are not mutually exclusive, as students indicated their expectation of majoring in each of the four fields. Among those planning to major in any STEM field, students were roughly split between those expecting to major in only one field (49%) and those expecting more than one (51%). Expectations to major in the biological and physical sciences are modestly correlated (R = 0.46), as are computer science and engineering (R = 0.34).

3 As 8th graders, students in the district took a survey or overview course covering topics in earth science, biology, and chemistry.

4 In analyses not shown here, we also included a measure of students’ science self-efficacy or self-confidence, as prior research finds that this positively predicts STEM outcomes [9]. However, because the survey only included one item to measure efficacy (“I usually do well in science”), and it did not significantly predict students’ plans to major in STEM net of their science affect, nor did it alter the impact the results shown here, we chose not to include it in the final models.

5 Multi-level random effects models yielded extremely similar results, but the variation across schools was not statistically significant.

6 Throughout, the estimated increases in probability of majoring in STEM are calculated using the *margins* post-estimation command in Stata.

7 Specifically, as students increase from one standard deviation below the mean to one standard deviation above the mean on the social relevance scale, their predicted probability of expecting to major in computer science increases from 0.24 to 0.29.

8 It is important to note that national studies have found that Hispanic as well as Black adolescents exhibit similar levels of interest in STEM fields as their white peers, and conditional on college matriculation, are as likely to enter STEM majors in college [48].

## Appendix A

**Table A1.** Zero-order correlations between variables.

	Expects to Major in STEM	Biological Sciences	Physical Sciences	Computer Science	Engineering	Female	Black	Hispanic	Born Outside of the U.S.	Books in the Home	Science Achievement	Science Affect	Perceived Social Relevance
Expects to major in STEM	1.00												
Biological sciences	0.41	1.00											
Physical sciences	0.41	0.46	1.00										
Computer science	0.54	0.13	0.19	1.00									
Engineering	0.66	0.07	0.18	0.34	1.00								
Female	−0.22	0.05	0.00	−0.16	−0.32	1.00							
Black	−0.02	0.00	−0.01	−0.02	−0.04	−0.01	1.00						
Hispanic	0.02	−0.01	−0.02	0.06	0.01	−0.02	−0.75	1.00					
Born outside of the U.S.	0.11	0.10	0.07	0.04	0.08	−0.03	−0.07	0.07	1.00				
Books in the home	0.07	0.10	0.08	−0.04	0.04	0.04	0.09	−0.31	0.01	1.00			
Science achievement	0.07	0.03	0.01	−0.03	0.10	−0.02	−0.06	−0.15	−0.07	0.31	1.00		
Science affect	0.18	0.17	0.21	0.10	0.10	−0.08	−0.01	−0.02	−0.02	0.07	0.10	1.00	
Perceived social relevance	0.11	0.15	0.15	0.07	0.06	0.03	0.11	−0.17	−0.04	0.11	0.21	0.40	1.00
